# Percutaneous Closure *vs.* Surgical Repair for Postinfarction Ventricular Septal Rupture: A Systematic Review and Meta-Analysis

**DOI:** 10.21470/1678-9741-2022-0417

**Published:** 2023-06-14

**Authors:** Xiangyang Wu, Cingting Wang, Xinyuan Du, Yongnan Li, Fengxiao He, Qiming Zhao, Yong Mao

**Affiliations:** 1 Department of Cardiac Surgery, Lanzhou University Second Hospital, Lanzhou University, Lanzhou, Gansu, People’s Republic of China; 2 Health Science Center of Lanzhou University, Lanzhou University, Lanzhou, Gansu, People’s Republic of China; 3 Tianjin University of Traditional Chinese Medicine, Tianjin, People’s Republic of China

**Keywords:** Myocardial Infarction, Ventricular Septal Rupture, Risk Factors, Meta-Analysis, Treatment Outocome

## Abstract

**Introduction:**

Ventricular septal rupture is an important high-mortality complication in the scope of myocardial infarctions. The effectiveness of different treatment modalities is still controversial. This meta-analysis compares the efficacy of percutaneous closure vs. surgical repair for the treatment of postinfarction ventricular septal rupture (PI-VSR).

**Methods:**

A meta-analysis was performed on relevant studies found through PubMed®, Embase, Web of Science, Cochrane Library, China National Knowledge Infrastructure (or CNKI), Wanfang Data, and VIP databases searching. The primary outcome was a comparison of in-hospital mortality between the two treatments, and the secondary outcome was documentation of one-year mortality, postoperative residual shunts, and postoperative cardiac function. Differences were expressed as odds ratios (ORs) with 95% confidence intervals (CIs) to assess the relationships between predefined surgical variables and clinical outcomes.

**Results:**

Qualified studies (742 patients from 12 trials) were found and investigated for this meta-analysis (459 patients in the surgical repair group, 283 patients in the percutaneous closure group). When comparing surgical repair to percutaneous closure, it was found that the former significantly reduced in-hospital mortality (OR: 0.67, 95% CI 0.48-0.96, P=0.03) and postoperative residual shunts (OR: 0.03, 95% CI 0.01-0.10, P<0.00001). Surgical repair also improved postoperative cardiac function overall (OR: 3.89, 95% CI 1.10-13.74, P=0.04). However, there was no statistically significant difference in one-year mortality between the two surgical strategies (OR: 0.58, 95% CI 0.24-1.39, P=0.23).

**Conclusion:**

We found that surgical repair appears to be a more effective therapeutic option than percutaneous closure for PI-VSR.

**Table:** 

Abbreviations, Acronyms & Symbols	
AMI	= Acute myocardial infarction
CABG	= Coronary artery bypass grafting
CI	= Confidence interval
CNKI	= China National Knowledge Infrastructure
CT	= Conservative treatment
LV-RA	= Left ventricular-right atrial
M-H	= Mantel-Haenszel
MI	= Myocardial infarction
NR	= Not reported
OR	= Odds ratio
PC	= Percutaneous closure
PI-VSR	= Postinfarction ventricular septal rupture
SR	= Surgical repair

## INTRODUCTION

Myocardial infarction (MI) is an acute condition with high morbidity and mortality rates throughout the world. Postinfarction ventricular septal rupture (PI-VSR), which has an incidence of 1% to 2%, is a rare but clinically fatal postinfarction complication^[1]^. Conservative medicinal therapy alone is only appropriate for patients with hemodynamically inconsequential defects or those whose surgical risk is prohibitive due to the high death rate associated with untreated defects. This can be close to 80% at 30-day postinfarction^[2]^.

Surgical repair is a common and established form of treatment, but it is extremely invasive and fraught with the possibility of residual shunts and recurrent perforation after the procedure. In patients with cardiogenic shock and respiratory failure, urgent PI-VSR surgical correction has been linked to a 40% death risk^[3]^. Patients with PI-VSR typically have a poor cardiac function and inadequate surgical trauma tolerance at the same time (especially those with poor physical fitness). With the advent of interventional techniques, percutaneous closure has become an additional therapy option for such patients. Although extracorporeal circulation difficulties, lengthy operations, and disturbance of the sternal structures are avoided with percutaneous closure, there is a chance that postoperative residual shunts and vascular issues will develop^[4,5]^.

Most importantly, there is still conflicting data on the efficiency of these two treatment options. To investigate this comparison further, we conducted a meta-analysis of the pertinent literature to compare the clinical results of percutaneous *vs.* surgical repair for the treatment of PI-VSR.

## METHODS

The components for this meta-analysis were reported using the Preferred Reporting Items for Systematic Reviews and Meta-Analyses (or PRISMA) statement, a 27-item checklist^[6]^. The research protocol has also been submitted to the International Platform of Registered Systematic Review and Meta-analysis Protocols (INPLASY2022100056).

### Search Strategy

The following seven electronic databases were comprehensively searched: Wanfang Data, VIP, China National Knowledge Infrastructure (or CNKI), Web of Science, Cochrane Library, PubMed®, and Embase. There were no restrictions set on the language or date of the literature search. The searches began on September 30, 2022. Studies detailed the results for patients over the age of 18 years who underwent percutaneous closure surgery or surgical repair for PI-VSR. The search was developed based on the PICOS principals, and search terms were “ventricular septal rupture” OR “ventricular septal ruptures” OR “ventricular septal perforation” OR “septal rupture, ventricular” OR “septal ruptures, ventricular” AND “surgery” OR “percutaneous closure surgery”. We manually searched reference lists of retrieved publications (including reviews) to find studies that might be eligible.

### Study Selection and Inclusion Criteria

All citations were exported into EndNote, and after removing duplicates, YM and XW evaluated the titles and abstracts considering the eligibility requirements (Table 1). To be included, only studies written in English were taken into consideration. For studies that, once reviewed, were found to be “included” or “uncertain”, full papers were obtained, and the publications were checked against the inclusion criteria again. Studies that had the most thorough data and had been consistently published were chosen for reporting. Any disputes over which studies should be chosen were settled through discussion, and a final decision was made by a third reviewer (CW).

**Table 1:** Meta-analysis’ inclusion and exclusion criteria.

	Inclusion criteria	Exclusion criteria
Language	English	Non-English
Publication dates	All years	
Participants	Age ≥ 18 years old	Age < 18 years old
	PI-VSR patients	Animal studies
		Not AMI-related ventricular septal rupture
Intervention	Surgical repair	Not according to the inclusion criteria
	Percutaneous closure surgery	
Study design	Randomized controlled trial	Case report
	Case control study	Review
	Cohort study	Protocol
		Commentary
		Letter
Outcome	In-hospital mortality	Data about mortality or another outcome not available
	One-year mortality	
	Postoperative residual shunt	
	Cardiac function grade (class I or II)	

AMI=acute myocardial infarction; PI-VSR=postinfarction ventricular septal rupture

### Data Extraction

Author, publication year, study design, interventions employed in the treatment or control groups, sample size, and meta-analysis results were all collected using a customized extraction form. The authors of these studies were not contacted for additional information.

### Quality Assessment

Two reviewers evaluated the quality of all included research independently (YM and XW). The quality of any nonrandomized controlled trials was evaluated using the Newcastle-Ottawa Scale (or NOS)^[7]^. Every included study was evaluated using the “star system”. A total score of 5 or less was regarded as poor, a score of 6 or 7 as moderate, and a score of 8 or 9 as high. Discrepancies were resolved by consultation and agreement between the other two reviewers (XD and CW). The primary outcome was a comparison of in-hospital mortality between the two treatments. The secondary outcomes are comparison results of one-year mortality, postoperative residual shunts, and postoperative cardiac function.

### Statistical Analysis

The Cochrane Collaboration’s Review Manager software version 5.3 was used for meta-analysis and Egger’s regression test. For dichotomous variables, the Mantel-Haenszel model was used to obtain odds ratio (OR) and 95% confidence interval (CI). Heterogeneity between studies was assessed by I2 statistics.

Values of 25, 50, and 75% were reported as low, moderate, and high degrees of heterogeneity, respectively. A subgroup analysis in the meta-analysis (focused on different study designs such as randomized controlled trials, prospective cohort studies, and retrospective studies) was conducted to lessen the heterogeneity. A *P*<0.05 was considered to be statistically significant. Egger’s regression model was used to detect publication bias when the number of studies analyzed was enough.

## RESULTS

**Fig. 1:**
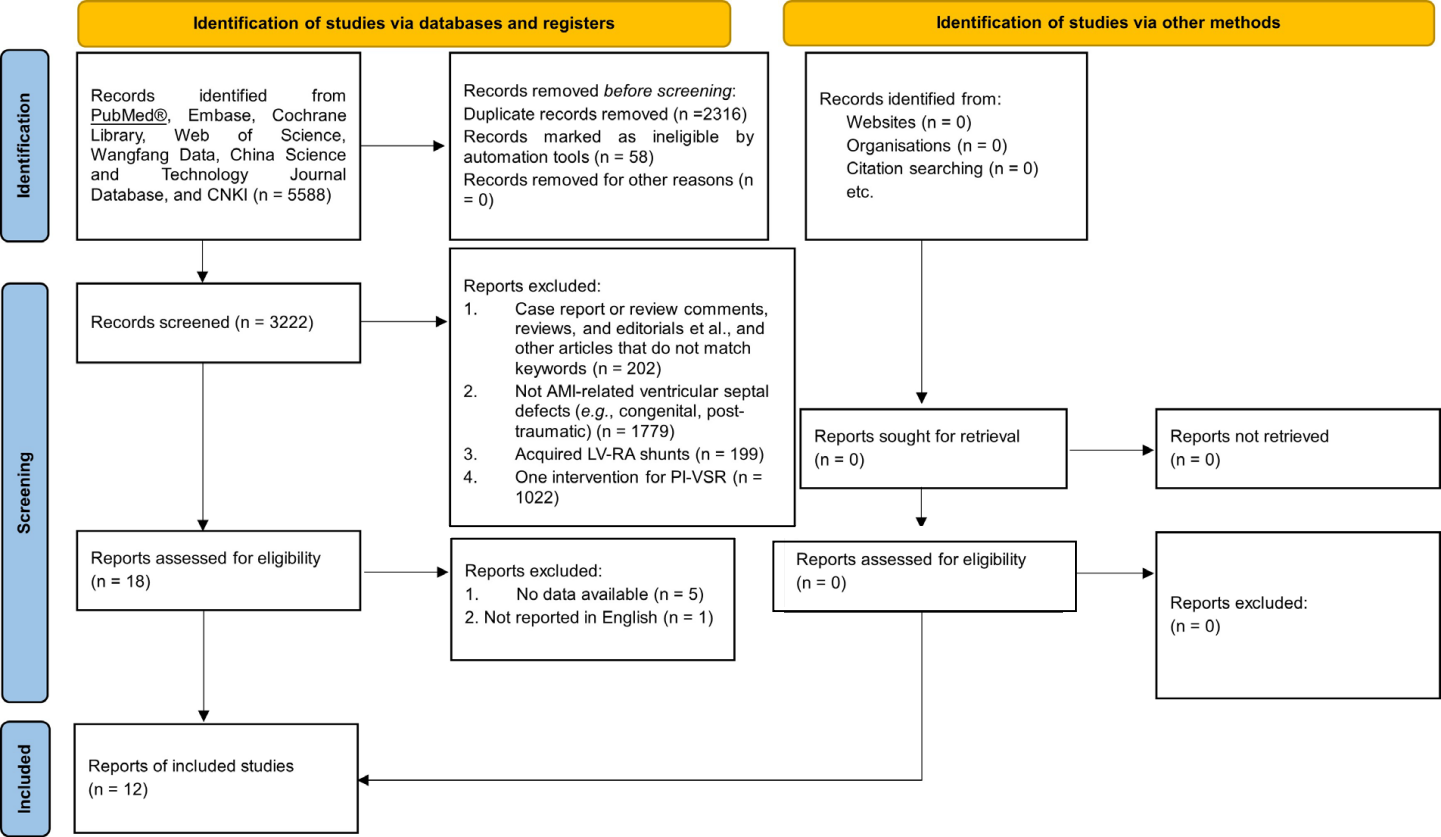
Preferred Reporting Items for Systematic Reviews and Meta-Analyses (or PRISMA) flowchart. AMI=acute myocardial infarction; CNKI=China National Knowledge Infrastructure; LV-RA=left ventricular-right atrial; PI-VSR=postinfarction ventricular septal rupture.

A summary of the study selection process is presented in Figure 1.

A total of 5,588 citations were found in the literature search. Of these, 2,374 research articles were eliminated for various reasons, including duplication. After reviewing the paper titles and abstracts, 3,024 articles were eliminated for PI-VSR or other reasons, depending on the type of article. Six papers were found to be invalid after the full-text versions of 18 publications were reviewed. Finally, 12 papers in total were down-selected and deemed suitable for analysis^[8-19]^.

### General Characteristics of the Included Studies

The key characteristics of the studies that fit the inclusion criteria are presented in Table 2. A total of 742 patients were included across these 12 papers, 459 of them fit the surgical repair group and 283 fit the percutaneous closure group. Six study groups within the papers analyzed compared surgical treatment with percutaneous closure. The other six groups of research evaluated surgical treatment, percutaneous closure, and conservative treatment. In terms of results, eleven of the studies looked at in-hospital mortality, three at one-year mortality, three at the residual shunt following surgery, and two at postoperative cardiac function.

**Table 2:** Description characteristics of the included studies.

Study	Study type	Age, years	Male, n	Patients number	Intervention comparison	Time from AMI to operation, days	Defect size, mm	Outcomes	Occluder brand	Newcastle- Ottawa Scale scores
Rojas-Velasco et al., 2011	Retrospective cohort study	64	27	40 (15/7/18)	SR vs. PC *vs.* CT	10.0 *vs.* 10.0	NR	①②④	Amplatzer	8
Yinjun et al., 2013	Retrospective cohort study	68	2	11 (7/4)	SR *vs.* PC	NR	NR	①	Shenzhen Lifetech (3) Amplatzer (1)	7
Kalyani et al., 2015	Retrospective cohort study	67	11	20 (14/6)	SR *vs.* PC	NR	NR	①	Amplatzer	8
Goldsweig et al., 2017	Retrospective cohort study	NR	54	102 (91/11)	SR *vs.* PC	NR	NR	②	NR	7
Yan et al., 2020	Retrospective cohort study	70.5	15	40 (3/16/11)	SR *vs.* PC *vs.* CT	NR	NR	①	NR	7
Xuewen et al., 2020	Retrospective cohort study	66.5	37	66 (22/18/26)	SR *vs.* PC *vs.* CT	31.6 *vs.* 20.4	13.0 *vs.* 13.0	①③	Shanghai Shape Memory Alloy	8
Xinyu et al., 2020	Retrospective cohort study	62.6	21	31 (18/13)	SR *vs.* PC	NR	NR	①②	NR	7
Bhattacharya et al., 2021	Retrospective cohort study	64.8	28	52 (22/5/25)	SR *vs.* PC *vs.* CT	NR	NR	①	Amplatzer	6
Yaguo et al., 2021	Retrospective cohort study	71	18	50 (5/16/29)	SR *vs.* PC *vs.* CT	14.0 *vs.* 19.0	10.6 *vs.* 13.1	①	NR	7
Dongliang et al., 2022	Retrospective cohort study	64.4	11	23 (17/6)	SR *vs.* PC	35.1 *vs.* 29.3	10.0 *vs.* 10.0	①③④	Shanghai Shape Memory Alloy	9
Giblett et al., 2022	Retrospective cohort study	68.8	289	362 (231/131)	SR *vs.* PC	9.0 *vs.* 9.0	18.0 *vs.* 20.0	①	Amplatzer (127) Occlutech (4)	8
Zhenzhen et al., 2021	Retrospective cohort study	68.1	48	110 (14/50/46)	SR *vs.* PC *vs.* CT	NR	24.3 *vs.* 11.9	①③	NR	8

① In-hospital mortality; ② one-year mortality; ③ postoperative residual shunt; ④ cardiac function AMI=acute myocardial infarction; CT=conservative treatment; NR=not reported; PC=percutaneous closure; SR=surgical repair

### Primary Outcome

#### In-hospital Mortality

**Fig. 2:**
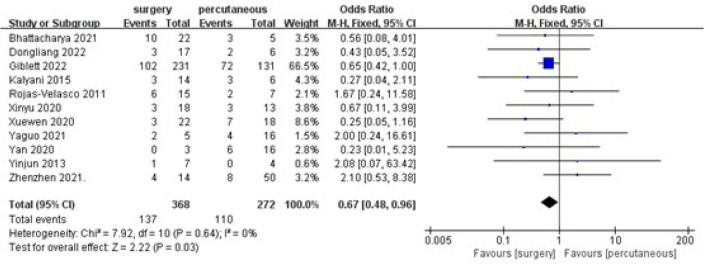
Comparing in-hospital mortality of surgical repair and percutaneous closure. CI=confidence interval; M-H=Mantel-Haenszel.

A statistically significant decrease was found when comparing in-hospital mortality in the surgical repair group to the percutaneous closure group (overall OR: 0.67, 95% CI 0.48-0.96, *P*=0.03) (Figure 2). Also, no heterogeneity was observed (I2=0%). The funnel plot is more symmetrical, suggesting less publication bias.

### Secondary Outcomes

#### One-year Mortality

**Fig. 3:**
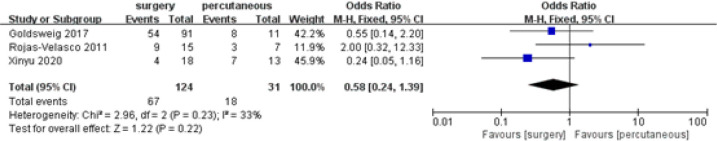
Comparing one-year mortality of surgical repair and percutaneous closure. CI=confidence interval; M-H=Mantel-Haenszel.

There were no significant differences observed in one-year mortality between the two therapy groups (overall OR: 0.58, 95% CI 0.24-1.39, *P*=0.23) (Figure 3). The groups showed only a moderate heterogeneity (I^2^=33%).

#### Postoperative Residual Shunt

**Fig. 4:**
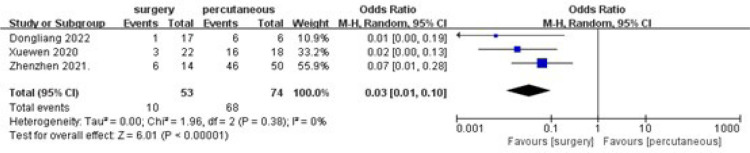
Comparing postoperative residual shunt of surgical repair and percutaneous closure. CI=confidence interval; M-H=Mantel-Haenszel.

A statistically significant decrease was found in the postoperative residual shunt frequency in the surgical repair group when compared to percutaneous closure (overall OR: 0.03, 95% CI 0.01-0.10, *P*<0.00001) (Figure 4). Also, no heterogeneity was observed (I^2^=0%).

#### Number of Postoperative Cardiac Function Grades (I or II)

**Fig. 5:**
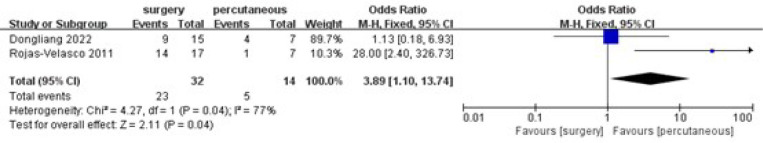
Comparing number of postoperative cardiac function grade of surgical repair and percutaneous closure. CI=confidence interval; M-H=Mantel-Haenszel.

A statistically significant increase was found in the number of postoperative cardiac function grades (I or II) in the surgical repair group when compared to percutaneous closure (overall OR: 3.89, 95% CI 1.10-13.74, *P*=0.04) (Figure 5). Also, high heterogeneity was observed in this group (I^2^=77%).

## DISCUSSION

PI-VSR is an infrequent life-threatening complication following MI. This meta-analysis compared the effectiveness of percutaneous closure with that of surgical repair for PI-VSR. This investigation demonstrated that surgical repair had better postoperative cardiac function, lower incidence of a residual shunt, and lower in-hospital mortality than percutaneous closure. However, there was no statistically significant difference in one-year mortality between the two surgical strategies.

The dominant right coronary artery or the dominant left circumflex artery are the main causes of posterior septal perforation. This perforation occurs in the proximal 1/3 of the septum. An anterior septal rupture is in the distal 2/3 of the septum and is primarily caused by MI through the anterior wall due to occlusion of the left anterior descending artery. In addition to coronary revascularization, surgical methods were used to treat PI-VSR. Patients who also had concurrent ventricular aneurysms underwent ventriculotomy or ventriculoplasty^[20]^. Even though the technique is more invasive, the lesion is completely relieved. The patient’s postoperative recovery of heart function is consequently better facilitated by this surgical technique^[21]^. In addition, the following characteristics were linked to the occurrence of greater residual shunts in the percutaneous occlusion group than in the surgical repair group: (1) the perforated ventricular septum is typically irregular in shape, and the occluder chosen is too small to cause residual shunts or too large to cause complications like atrioventricular block and ventricular arrhythmias; (2) the tissue of the perforated ventricular septum after MI is brittle and there may be small gaps between the septum and the occluder after blocking, some of which may form after the blocking procedure; (3) the location of the defect following a severe inferior wall MI is frequently on the free wall at the base of the right and left ventricles, which has an impact on the occluder disc’s deployment^[22-25]^.

The effectiveness of percutaneous intervention and surgical repair have not been previously compared in a meta-analysis. The effectiveness of this specific PI-VSR therapy modality has been carefully assessed by several researchers. Flynn^[26]^ included 314 patients who underwent percutaneous occlusion of the PI-VSR in 25 trials, with an in-hospital mortality rate of 37.5%. Matteucci^[27]^ included 6,361 patients in 41 studies with a surgical mortality rate of 38.2%. The surgical and percutaneous intervention groups each had an in-hospital death rate of 37.2% and 40.4%, totaling 742 patients, which was comparable to the outcomes of the two systems analyzed above. Furthermore, Ronco’s systematic^[28]^ analysis found no statistically significant difference between contemporaneous coronary artery bypass grafting (CABG) and no CABG in the management of mechanical complications after MI in terms of immediate or long-term mortality. The magnitude of the PI-VSR defect was not related to death, according to Yang’s^[29]^ study.

### Limitations

This meta-analysis has several limitations. First, the included studies were all retrospective, and it was not possible to control potential confounding factors. Second, the use of various occluder brands could be an additional confounding factor. Finally, different intervention times could also be a confounding factor.

## CONCLUSION

Acute MI complications like PI-VSR are uncommon, yet deadly. We conducted a meta-analysis and concluded that, for PI-VSR, surgery seems to be a safer therapeutic choice than percutaneous closure. In the future, large-scale randomized controlled trials are required to confirm the effects of percutaneous closure and surgical repair.

**Table:** 

Authors’ Roles & Responsibilities	
XW	Substantial contributions to the conception and design of the work; and the analysis and interpretation of data for the work; drafting the article and revising it; final approval of the version to be published
CW	Substantial contributions to the acquisition, analysis, and interpretation of data for the work; revising the work critically for important intellectual content; final approval of the version to be published
XD	Substantial contributions to the acquisition, analysis, and interpretation of data for the work; revising the work critically for important intellectual content; final approval of the version to be published
YL	Substantial contributions to the analysis and interpretation of data for the work; revising the work critically for important intellectual content; final approval of the version to be publishe
FH	Substantial contributions to the acquisition and analysis of data for the work; revising the work critically for important intellectual content; final approval of the version to be published
QZ	Substantial contributions to the acquisition and analysis of data for the work; revising the work critically for important intellectual content; final approval of the version to be published
YM	Substantial contributions to the conception and design of the work; and the acquisition, analysis, and interpretation of data for the work; drafting the work and revising it; final approval of the version to be published
